# The dark-side of the outside: how extracellular heat shock proteins promote cancer

**DOI:** 10.1007/s00018-021-03764-3

**Published:** 2021-02-05

**Authors:** Laura Seclì, Federica Fusella, Lidia Avalle, Mara Brancaccio

**Affiliations:** Department of Molecular Biotechnology and Health Sciences, University of Torino, Turin, Italy

**Keywords:** Extracellular, Chaperones, Heat shock proteins, Tumour microenvironment, Cancer hallmarks

## Abstract

In addition to exerting several essential house-keeping activities in the cell, heat shock proteins (HSPs) are crucial players in a well-structured molecular program activated in response to stressful challenges. Among the different activities carried out by HSPs during emergency, they reach the extracellular milieu, from where they scout the surroundings, regulate extracellular protein activity and send autocrine and paracrine signals. Cancer cells permanently experience stress conditions due to their altered equilibrium and behaviour, and constantly secrete heat shock proteins as a result. Other than supporting anti-tumour immunity, extracellular heat shock proteins (eHSPs), can also exacerbate cancer cell growth and malignancy by sustaining different cancer hallmarks. eHSPs are implicated in extracellular matrix remodelling, resistance to apoptosis, promotion of cell migration and invasion, induction of epithelial to mesenchymal transition, angiogenesis and activation of stromal cells, supporting ultimately, metastasis dissemination. A broader understanding of eHSP activity and contribution to tumour development and progression is leading to new opportunities in the diagnosis and treatment of cancer.

## Introduction

“It did not matter if this interpretation was true or false, it was a working link between imagination and reality, like love” [1].

This is how Ferruccio Ritossa described his discovery of the heat shock proteins, and at least 20 years later, this perception is still alive, considering the finding of new and unexpected roles played by these multifaceted proteins.

Heat shock proteins (HSPs) were originally identified as stress-responsive proteins required for cell survival during thermal stress, acting as molecular chaperones. Shortly afterwards, it became clear that HSPs are induced in response to a wider variety of insults (of physical, chemical and biological origin), preventing cell death. However, despite the name, most HSPs are ubiquitously expressed even in physiological conditions, since they are essential for housekeeping functions inside the cell [2]. Cells possess different families of chaperones with specific activities and functions, often working in cooperation to fold native proteins and assist the formation of supramolecular complexes, keep proteins in activation-competent conformations, and stabilize them during the conformational changes required for their activities. Chaperones are also required to re-fold denatured polypeptides, inhibit unfolded protein aggregation, and, when proteins are defective or irreversibly misfolded, direct them to degradation via proteasomal and autophagic pathways. To exert their functions on their substrate proteins, named clients, HSPs typically take part in complexes that contain other chaperones, co-chaperones, modulators of ATPase activity and various accessory proteins. A clear nomenclature for the HSPs and related chaperone genes was proposed in 2009 [3]; HSPs are currently classified according to their size into six major and broadly conserved families HSP100s, HSP90s, HSP70s and HSP60s, that use ATP hydrolysis to carry out their activity, HSP40s and small heat shock proteins (sHSPs), whose do not possess ATPase activity per se.

## Some HSPs are secreted into the extracellular milieu

**Fig. 1:**
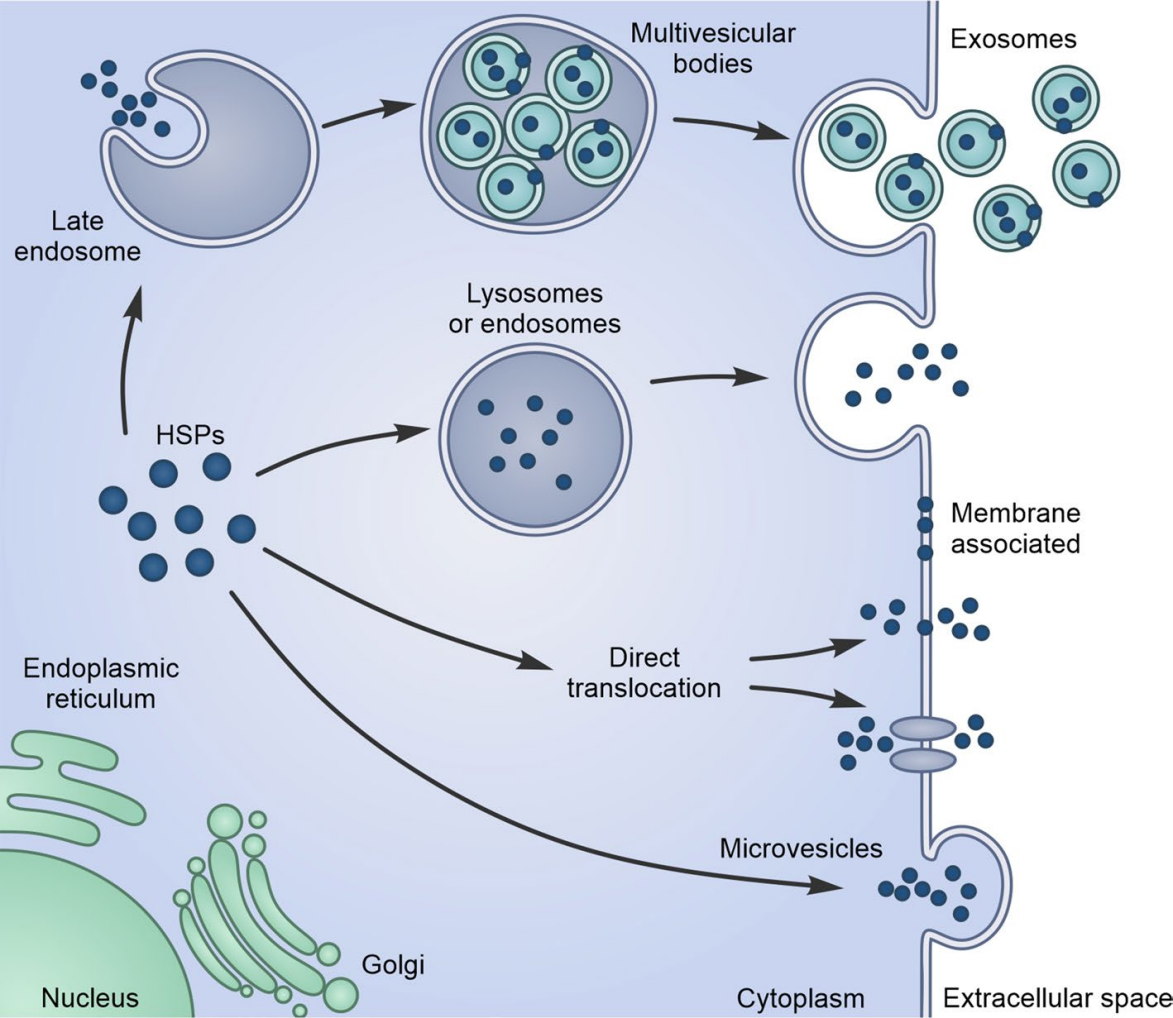
Pathways of unconventional Heat Shock Proteins (HSPs) secretion in the extracellular space. Lysosomes or endosomes fusion with plasma membrane leads to the release of HSPs in the extracellular space. HSPs can be captured from the cytoplasm during the formation of endosomal internal vesicles, which leads to the biogenesis of multivesicular bodies. These internal vesicles are then released as exosomes. HSPs can directly translocate from the cytoplasm across the plasma membrane facilitated or not by ATP-binding cassette (ABC) transporters. Microvesicle shedding from the cell surface can also lead to the release of HSPs into the extracellular space

**Fig. 2:**
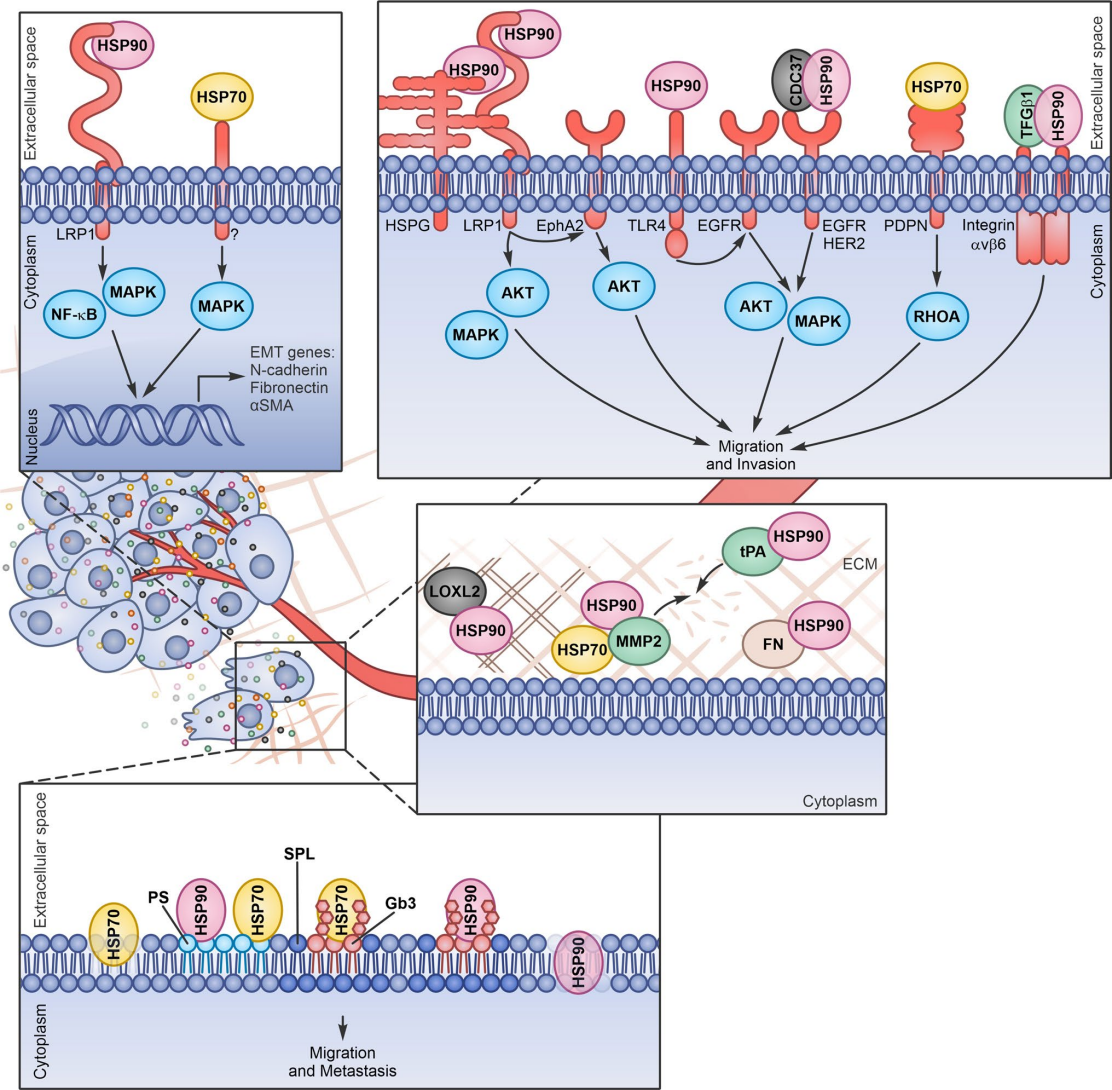
Extracellular Heat Shock Proteins (eHSPs) induce extracellular matrix (ECM) remodelling, epithelial–mesenchymal transition (EMT), migration and invasion. eHSPs released by cancer cells can interact with extracellular client proteins favouring ECM remodelling and with surface receptors, triggering signal transduction inside the cells (*HSPG* heparan sulfate proteoglycans, *FN* fibronectin, *PDPN* podoplanin)

The year was 1986 when Tytell [4] described for the first time the transfer of HSPs from glia to axon; almost at the same time, another article by Hightower and Guidon [5] showed the release of HSPs from cultured rat embryo cells underlying their uncanonical mechanism of secretion. For many years, these results did not convince the scientific community and were considered as potential artefacts caused by cell necrosis [6]. One of the issues questioning the active release of HSPs was the absence of a secretion leader signal in their sequence [7]. Finally, robust data demonstrating the active secretion of HSPs by living cells were provided and the scientific community fully accepted this evidence [8, 9]. However, passive release from damaged or dead cells has been also observed in many cases [8, 9]. The lack of involvement of the canonical secretory pathway in HSP secretion was clearly demonstrated using inhibitors of endoplasmic reticulum (ER)-Golgi vesicular trafficking. Like interleukin (IL) 1 α/β and fibroblast growth factor (FGF), HSPs use alternative mechanisms for secretion [10, 11] including free release, vesicles or vesicular intermediates, derived from the autophagic membranes, endosomes and possibly secretory lysosomes [9] (Fig. 1). Even if the molecular details at the basis of eHSP secretion are not completely elucidated, what is clear is that many mechanisms could subsist and coexist for the same HSP in the same cell. For example, HSP70 secretion as free molecule is mediated by transmembrane proteins like ATP-binding cassette (ABC) transporters but could also occur via translocation through plasma membrane, as described for FGF2 [7]. Moreover, HSP70 requires the ABC family transporter proteins also to enter the endosomal or lysosomal vesicles, which in turn are secreted in the extracellular compartment [7, 12, 13]. Another mechanism is mediated by exosomes, where HSPs amount increases when cells are under heat-shock or other stress conditions. HSPs contained in exosomes are transferred to target cells modifying their behaviour [14]. eHSPs localized on the membrane of exosomes can also engage surface receptors and trigger intracellular signalling in an autocrine or paracrine fashion [15–20]. eHSPs localizes within the lipid bilayer of cellular plasma membranes by interacting with membrane lipids, possibly stabilizing membranes, regulating their physical properties and organizing microdomain composition [21] (Fig. 2). For example, intracellular HSP70, recognizing phosphatidylserine (PS) moieties, phospholipids that are normally present in the cytosolic side of cellular membranes, undergoes an insertion into the lipid bilayer, exposing a small region of its C-terminus end to the extracellular environment. It has been proposed that, during the recovery condition after a stress stimulus, HSP70 accumulates in respect to unfolded proteins, oligomerizes and binds to PS moieties, being embedded in the plasma membrane. In addition, the increase in HSP70 plasma membrane localization is associated with the formation of ion conductance pathways, resulting in cell death. However, some reports suggest that HSP70 interaction with lipid bilayer lies also on cholesterol-rich microdomains, regions within the plasma membrane that are enriched not only in cholesterol but also in glycosphingolipids, glycosylphosphatidylinositol-anchored and acetylated proteins [21–24]. Experimental data indicate that HSP70 is exposed on the surface of tumour cells through the binding with the ceramide-derived glycosphingolipid globotriaoslyceramide (Gb3) that accumulates in lipid rafts [25].

## Extracellular HSPs and cancer

HSPs levels have been found aberrantly high in human cancers compared to normal tissues and correlated with poor prognosis [26, 27]. HSPs overexpression in cancers is a response to the internal stress experienced by malignant cells, such as lack of proteostasis due to high levels of protein synthesis and to the presence of mutant proteins, and to stresses imposed by the hostile tumour microenvironment (TME), like hypoxia, nutrient deprivation, and acidosis [28]. Cancer growth is a complex, multistep process that requires cells to acquire some intrinsic characteristics, as genomic instability, resistance to cell death, altered metabolism and motility and to modify the TME inducing angiogenesis, inflammation and immune evasion [29, 30]. The role of intracellular chaperones and co-chaperones in sustaining transformation and cancer progression is well known, as the attitude of cancer cells to become addicted to HSP overexpression [31]. It is now widely accepted that HSPs are released by different types of cancers and are associated with their plasma membranes [32]. Several studies describe the tumour-specific immunogenicity of these released or surface-localized HSPs, a function associated with their ability to chaperone antigenic peptides and to activate anti-tumour innate immunity [33, 34]. The role of eHSPs in inhibiting inflammation and in promoting immune surveillance has been extensively investigated and recently reviewed [35–39], and therefore is not subject of debate in this review.

But, despite their tumour-alerting role, the surface and extracellular localization of HSPs may not be entirely beneficial to the host and, on the contrary, can play key functions in promoting tumour progression and metastasis formation, modulating several cancer hallmarks [38, 40–42]. Different extracellular chaperones and co-chaperones have been reported to be secreted by cancer cells; however, the majority of data demonstrating an active role of eHSPs in cancer refers to HSP90, HSP70 and HSP27.

HSP90 is the most abundantly expressed protein accounting for the 2–3% of the total proteins in normal cells and up to 7% in tumour cells [43]. HSP90 comes in two isoforms: the inducible HSP90α and the constitutive HSP90β. While HSP90β, but not HSP90α, is critical for the cell viability, HSP90α is mainly involved in cell responses to external stressors. Of note, cancer cells predominantly secrete the HSP90α isoform [44]. Indeed, analysing the sera of patients with cancer (including esophageal squamous cell carcinoma, melanoma, lung, breast, liver, pancreas and prostate cancer), eHSP90α level positively correlates with tumour progression [45, 46] as well as with the metastatic lesions in distant organs [46–48], functioning as a useful diagnostic and prognostic biomarker. eHSP90α translocation in the plasma membrane of different cancer cells and its subsequent release in the medium relies on different stimulus-activated cascades as the hypoxia-inducible factor (HIF) 1α and the epidermal growth factor (EGF)-induced phospholipase (PLC) γ1/protein kinase C (PKC) γ signalling [49]. Moreover, eHSP90α secretion in breast cancer cells depends also on protein modifications, including PKA-mediated phosphorylation [48] and acetylation [50]. The HSP70 family includes several slightly different proteins: among them, the constitutively expressed HSC70 and the stress-inducible HSP72, the HSP70 located in the mitochondria (GRP75) and GRP78, resident in the endoplasmic reticulum. Cell membrane localization of eHSP70 has been found on different cancer cell lines and on patients’ tumours and metastases, but not on the normal tissues counterparts [51]. In particular, eHSP72 is described on the surface of sarcoma, lung carcinoma and pancreatic cancer cell lines [52–54]. eHSP70 has been found on the surface of cancer cells in metastatic lesions in melanoma patients, together with eHSP90 [45, 51]. Exosomes released by cancer cells expose HSP70 on their membranes, while exosomes derived by normal cells do not. This feature makes the presence of HSP70 on patients’ exosomes a promising biomarker for monitoring cancer growth, relapse and appearance of metastasis, useful to guide therapeutic interventions [52, 55–58]. eHSP27, a member of the small HSPs, has been found in the sera of patients with squamous cell carcinoma of the tongue, breast, liver and ovarian cancer [59–64] and its secretion is associated with enhanced tumour growth and metastasis.

After release in the extracellular compartment or inside vesicle membranes, HSPs can chaperone and activate specific extracellular client proteins or directly interact with surface receptors, unleashing specific intracellular signals in target cells. In both cases, eHSPs have been described to promote malignancy and enhance metastasis formation.

### Extracellular client proteins in cancer

Extracellular matrix (ECM) is the non-cellular constituent of tissues that provides both biochemical and structural support for the cellular component, favouring cell–cell communication, cell adhesion, and cell proliferation. In addition to water and minerals, it is composed of collagen, proteoglycans, laminin, and fibronectin secreted by resident cells. ECM is a highly dynamic structure, constantly undergoing a remodelling process, in which components are degraded and modified by different proteases. Of note, the majority of the eHSP client proteins are ECM or enzymes involved in ECM remodelling [65–69]. ECM remodelling is a hallmark of cancer progression, impacting on cell proliferation, migration, and apoptosis [70, 71]. Indeed, a common feature of cancer malignancies is the excessive production of collagens, which are the major components of the ECM. Collagens can act as a scaffold, facilitating migration of invading cancer cells or stromal cells. In addition, increased collagen deposition and increased fibril cross-linking is associated with major changes in biomechanical properties of tissues, contributing to stiffness, which is crucial for a tumour to displace the host tissue and grow in size [72].

Fibronectin is secreted by cells as a soluble dimer and the subsequent binding to integrin receptors, exposed on the cell surface, induces a fibronectin conformational changes that expose self-association domains and promote the formation of an insoluble matrix in the extracellular space. Increased turnover of the extracellular fibronectin matrix has been correlated with enhanced metastatic capacity of tumour cells. As collagen assembly relies on fibronectin, fibronectin network depends on collagen and together can favour migration and invasion of both cancer cells and activated fibroblasts [70, 71].

ECM modifying enzymes such as matrix metalloproteinases (MMPs), heparanase, cathepsins, plasminogen activator (PA) and the lysyl oxidase (LOX) family are in charge of ECM structuring and turnover and their deregulated expression in tumours, significantly contribute to cancer progression and metastasis. Of note, a number of these enzymes depend on eHSPs for their activity and stability.

MMPs are structurally related zinc metalloproteinases, present on the cell surface or secreted in the extracellular compartment. At present, more than 21 mammalian MMPs have been identified and they are historically classified according to their substrate specificity and structural similarity in collagenases, gelatinases, stromelysins and matrilysins [73]. Due to the increasing number of proteins identified, MMPs are now divided into eight groups according to their structure. The MMPs are synthesized as inactive zymogens (pro-MMPs) and their activation requires proteolytic removal of the prodomain [73]. Most of the MMPs are activated outside the cell by other activated MMPs or serine proteinases. MMP2 and MMP9 are frequently overexpressed and highly secreted in human cancers. These soluble gelatinases, whose preferential substrates include type IV collagen, elastin, vitronectin, and aggrecan, possess also non proteolytic activities, regulating signalling pathways that control cell growth, inflammation, or angiogenesis [74, 75].

The plasminogen activator is a serine protease that cleaves the inactive proenzyme plasminogen into active plasmin; a broad spectrum serine protease that is, in turn, able to degrade fibronectin, laminin, vitronectin, proteoglycans, as well as fibrin and activate latent collagenases, including MMPs. Plasminogen activation is catalysed by urokinase-type (uPA) or tissue-type (tPA) plasminogen activators, which are subjected to time- and space-dependent regulation. In particular, the role of PAs in tissue remodelling seems consistent with the finding of their overexpression in human tumours, as well as their elevated amount in the plasma of breast, prostate, head and colon cancer patients [70].

Lysyl oxidases (LOX) are secreted amine oxidases. The family includes five members (LOX and LOX-like 1–4), whose primary function is the covalent crosslinking of collagens and/or elastin in the ECM. The aberrant expression, secretion and activity of these proteins have been reported in a range of human cancers. Indeed, some LOX members (in particular LOXL2) promote tumour cell survival, regulate cell adhesion, motility and invasion, and remodel the TME. LOX- and LOXL2-mediated tumour progression is due primarily to ECM modifications but relies in part on intracellular signalling. Upregulation of LOXL2 has been observed in a number of human cancers, and its expression has been associated with cancer aggressiveness [70, 76].

### eHSP receptors in cancer

In addition, eHSPs can directly interact with several cell surface receptors influencing cell behaviour through both autocrine and paracrine signalling [77]. The main HSP-activated receptors involved in cancer progression are Low density lipoprotein receptor-related protein 1 (LRP1), Toll Like Receptors (TLRs), the EGF receptor family (ERBB) and cluster of differentiation 40 (CD40) [78, 79].

LRP1 or CD91 protein consists of a large extracellular ligand-binding subunit non-covalently associated to a smaller subunit, containing a transmembrane domain and a short cytoplasmic tail. LRP1 is expressed in a large panel of cells, such as hepatocytes, fibroblasts, smooth muscle cells, neurons and astrocytes [80]. More than forty LRP1 ligands have been identified nowadays, including apolipoproteins, proteinases, proteinase-inhibitor complexes, bacterial toxins, viruses, the blood coagulation factor VIII, and various extracellular matrix proteins such as MMPs and uPA [81]. LRP1 is internalized in vesicles to deliver bound ligands to the endosomal/lysosomal compartment and is then recycled on the plasma membrane [82]. Beyond its ability to internalize extracellular components, LRP1 initiates and regulates many signalling pathways, as small Rho family GTPases, extracellular signal-regulated kinase (ERK), AKT and c-Jun N-terminal kinase (JNK) pathways [83–85]. Indeed, LRP1 expression is often deregulated in human cancers. LRP1 also exerts a fundamental role in cytoskeleton organization, focal adhesion disassembly and integrin β1 maturation, regulating cell adhesion, spreading, migration and invasion. It was proposed for the first time as the receptor of several extracellular chaperones by the group of Srivastava [86] and later many other studies confirmed its role as eHSP receptor in cancer cells.

TLR family members are type I transmembrane glycoproteins structurally characterized by the presence of a leucine-rich repeat domain in their extracellular region and a Toll/IL-1 receptor (TIR) domain in their intracellular portion, which activates common signalling pathways via TLR-specific adaptor proteins. [87, 88]. To date, ten TLRs have been identified in humans, the expression of which has been demonstrated on various innate immune cells, such as macrophages, neutrophils, and dendritic cells (DCs), as well as non-immune cells including epithelial and endothelial cells. While, TLRs 1, 2, 4, 5 and 6 are expressed on the cell surface, TLRs 3, 7, 8 and 9 are found almost entirely within endosomes [89]. Although individual TLRs recognize distinct ligands, the mechanisms of TLR activation and signal transduction are highly conserved and involve MyD88-dependent and -independent pathways that, in turn, activate multiple pro-inflammatory signalling cascades including nuclear factor kappa-light-chain-enhancer of activated B cells (NF-κB), JNK/activator protein 1 (AP1), ERK and p38 [87, 88]. The most common ligands of TLRs are the bacterial cell-surface lipopolysaccharides (LPS), lipoproteins and lipopeptides of bacterial origin, proteins such as flagellin from bacterial flagella, double-stranded RNA of viruses and the unmethylated CpG islands of bacterial and viral DNA. It was shown that TLRs (in particular TLR2 and 4) can be also activated by many endogenous molecules including fibrinogen, surfactant protein-A, heparin, β-defensin 2, High Mobility Group Box 1 (HMGB1) and HSPs. In particular, TLR2 and 4 have been extensively validated as eHSP receptors and some indications exist also for TLR3 (when present in plasma membrane [89]) and TLR5 [87]. TLRs may promote carcinogenesis by unleashing pro-inflammatory, anti-apopototic, proliferative and pro-fibrogenic signals on tumour cells and cells of the tumour microenvironment, as fibroblasts, immune and endothelial cells [88].

ERBB receptors are a subclass of the receptor tyrosine kinase superfamily and comprises four members: EGFR/ERBB1, human epidermal growth factor receptor (HER)2/ERBB2, HER3/ERBB3 and HER4/ERBB4. All members have an extracellular ligand-binding region, a single membrane-spanning region and a cytoplasmic tyrosine-kinase-containing domain. The ERBB receptors are expressed in various tissues of epithelial, mesenchymal and neuronal origin and their ligands are members of the EGF family of growth factors. Activated ERBBs stimulate many intracellular signalling pathways including the mitogen-activated protein kinase (MAPK) and the phosphoinositide 3-kinase (PI3K)/AKT pathways. Despite extensive overlap in the molecules that are recruited to the different active receptors, different ERBBs preferentially modulate certain signalling pathways, owing to the ability of individual ERBBs to bind specific effector proteins. These receptors are implicated in the development of many types of tumours favouring proliferation, apoptosis inhibition and cancer progression [90].

CD40 is a member of the tumor necrosis factor (TNF) receptor superfamily, expressed by B cells, dendritic cells, monocytes, platelets and macrophages as well as by non-hematopoietic cells such as myofibroblasts, fibroblasts, epithelial and endothelial cells. CD40 ligand (CD40L) is a member of the TNF superfamily that binds to CD40 promoting the activation of signal transduction pathways thanks to several TRAF (TNF Receptor Associated Factor) proteins, including TRAF1, TRAF2, TRAF3, TRAF5, and TRAF6. CD40-mediated signalling pathways include the activation of NF-κB, MAPK and signal transducer and activator of transcription (STAT) 3, favouring the generation of an acquired immune response. In particular, in cancer, CD40 can license DCs to promote anti-tumour T cell activation and re-educate macrophages, from M2 state to M1 state, leading to fibrosis degradation and tumour regression [91, 92].

## eHSPs tune cancer hallmarks

### Extracellular matrix remodelling

eHSPs regulate ECM remodelling and stiffness interacting and regulating several extracellular client proteins. eHSP90α (and in some cases HSP70) has found to be crucial for the invasiveness and metastasis formation of fibrosarcoma, esophageal squamous cell carcinoma and breast cancer cells. eHSP90α assists the proteolytic activation of MMP2 in conjunction with HSP70 and the co-chaperones Hop, HSP40, and p23 and protects MMP2 from inactivation, covering one of its autocatalytic cleavage site [46, 48, 65, 68]. eHSP90α acetylation seems crucial to facilitate its association with MMP2 in the extracellular contest [50]. In addition, LOXL2 interacting with eHSP90α, reaches its functional conformation and induces migration in breast cancer cells [66]. Moreover, eHSP90α is found associated with the tPA in the medium of fibrosarcoma and breast cancer cells. This interaction is essential to convert and activate plasminogen to plasmin, favouring invasion [67]. eHSP90α and eHSP90β are found on the surface of invasive cancer cells, accumulated on the leading edge and then released in the extracellular space. Invadopodial protrusion formation is the first step in tumour invasion and eHSP localization and activity in this site probably depend on their ability to interact with specific ECM proteases that have found to be essential for the matrix degradation activity of invadopodia [49, 68, 93–95]. eHSP90α and eHSP90β are found in a common complex with fibronectin on the surface of breast cancer cells. In particular, eHSP90β more than eHSP90α binds to fibronectin, favouring its stability and protecting it from degradation, thus participating in its assembly and turnover. eHSP90 maintains the stability of fibronectin, and when the chaperone is inhibited, fibronectin is internalised and degraded in lysosomes in breast cancer cells. However, despite the observed interaction between eHSP90β and fibronectin, it is also possible that fibronectin assembly or turnover relies on the indirect activity of eHSP90 clients. In addition, fibronectin and eHSP90 have been found to co-localize in a common complex with LRP1 on the surface of breast cancer cells. After the inhibition of HSP90, LRP1 is the putative receptor that mediates the clearance of fibronectin, but the exact mechanism remains to be elucidated. Indeed, it has been previously reported that LRP1 is able to mediate fibronectin internalization and degradation following its accumulation on fibroblasts surface. It is also possible that fibronectin internalization mediated by LRP1 is the result of specific signalling activated by eHSP90. Indeed, AKT and NF-κB activation (eHSP-LRP1 mediated signalling pathways) has been reported to be involved in fibronectin turnover [69, 96, 97].

### Epithelial-mesenchymal transition

The epithelial-mesenchymal transition (EMT) process is a crucial cancer hallmark that involves the disruption of cell–cell adhesion and cellular polarity, remodelling of the cytoskeleton, and changes in cell–matrix adhesion, improving migratory and invasive properties. Cytokines, growth factors and eHSPs secreted in the TME, by interacting with cancer cell plasma membrane receptors, may induce intracellular signalling pathways that favour EMT [98]. In prostate cancer cells, eHSP90 binds to LRP1 and promotes ERK signalling, leading to the impairment of E-cadherin function, the loss of junctional integrity and the induction of EMT [99, 100]. eHSP90 is found to upregulate a cohort of stem-associated markers in prostate cancer cells, promoting self-renewal and stemness associated with metastatic propensity [101]. In colorectal cancer (CRC) cells, eHSP90α through LRP1, increases the levels of phosphorylated IκB kinase (IKK) α/β and NF-κB and induces the expression of TCF12, a class I member of the helix-loop-helix protein family preferentially overexpressed in CRC patients with cancer metastasis. TCF12 is responsible for eHSP90α-dependent fibronectin expression and the repression of E-cadherin, connexin-26, connexin-43, associated with the EMT program [102]. In liver cancer cells, eHSP70, activating p38/MAPK signalling pathway through an unknown receptor, causes E-cadherin reduction and alpha smooth muscle actin (αSMA) overexpression favouring EMT, migration and invasion [103]. An alternative mechanism by which eHSPs may induce EMT in breast cancer relies on the ability of the eHSP client proteins MMP2 and MMP9 to proteolytically cleave and activate latent transforming growth factor (TGF) β [104].

### Resistance to apoptosis

In several pathological situations, including cancer, different stimuli can induce eHSP secretion in the extracellular milieu, where these chaperones support the response to the stress insult protecting cells from apoptosis [6]. In particular, eHSP90 once secreted under hypoxia, induces the activation of AKT pathway, through the binding to LRP1 receptor, and seems essential to protect breast cancer cells from hypoxia-triggered death [105]. In line with this, in glioblastoma (GBM) cells, hypoxia not only amplifies eHSP90α secretion and its signalling, but also enhances LRP1 expression resulting in a positive loop that fuels cancer survival and progression [106]. Moreover, eHSP70 localization on the surface of colon carcinoma cells increases after radiotherapy and is associated with cell survival [107]. In a hepatocarcinoma model, eHSP72 binding to TLR2 and TLR4, promotes apoptotic resistance to chemotherapy and induces proliferation. In this study eHSP72, inducing the release of HMGB1 from cancer cells, favours also a long-lasting effect of TLR4 signalling, supporting tumour growth [108]. eHSP27 is also able to induce resistance to apoptosis. Once released from tongue cancer cells following chemotherapy, eHSP27 binds to TLR5 and triggers NF-κB signalling to enhance chemoresistance and promote cancer progression both in vitro and in vivo. In addition, the treatment with neutralizing antibodies against HSP27 and HSP70 sensitizes cancer cells, respectively to chemotherapy and radiotherapy [59, 107].

### Migration and invasion

HSP90α, by binding to LRP1 and activating ERK and AKT pathways, induce migration and invasion in breast cancer cells [43]. In GBM, the eHSP90α/LRP1 signalling is required to sustain AKT activation and the AKT-dependent phosphorylation of EphA2 (S897), a tyrosine kinase receptor that is overexpressed in the majority of GBM. EphA2 functions as an LRP1 co-receptor and its phosphorylation on S897 is crucial for its interaction with LRP1. The signalling facilitates lamellipodia formation, supporting GBM cell motility and invasion [106].

eHSP90, together with its co-chaperone CDC37, also interacts with the family of ERBB receptors, including EGFR or HER2, to promote cancer cell motility and invasion. The binding of eHSP90 to HER2 induces its heterodimerization with HER3, which in turn activates the MAPK and PI3K-AKT pathways, leading to actin rearrangement necessary for cell motility. eHSP90-dependent cancer cell migration is impaired by a monoclonal antibody, mAb 4C5, able to disrupt the interaction between CDC37 and HSP90 and CDC37 and ERBB [109, 110]. mAb 4C5 has been proved effective also in inhibiting invasion and metastasis dissemination in a preclinical model of melanoma [111]. eHSP90α has also been described to transactivate EGFR, another member of ERBB family, favouring GBM cell migration. More specifically, eHSP90α triggers TLR4, which in turn, leads to the phosphorylation and activation of EGFR in a PKCδ/proto-oncogene tyrosine-protein kinase (c-Src)-dependent manner, favouring calcium mobilization and ATP release, events known to be associated with cell migration [112]. Aside from eHSP90α, there are also some reports that describe a pro-tumorigenic role of eHSP90β on primary colon adenocarcinoma cells and on their lymph node metastasis-derived counterpart. In this context, eHSP90β binds to TGFβ1 forming a complex that, instead of binding TGFβ1 canonical receptors to activate the Smad2/3 signalling pathway, engages the integrin αvβ6 and promotes cancer cell invasion and metastasis through a pathway still undetermined [113].

In hepatocellular carcinoma and lung cancer cells, HSP70 promotes cancer progression by binding to TLR2 and to the receptor for advanced glycation end products (RAGE) and inducing MyD88-dependent and -independent NF-κB activation and pro-inflammatory gene transcription [108, 114]. GRP75, the member of the HSP70 family predominantly localized in the mitochondria, is also secreted by cancer cells. In particular, GRP75 and podoplanin, a mucin-type transmembrane sialoglycoprotein, are able to regulate the activities of Rho, ezrin, and other proteins linked to the actin cytoskeleton, co-localize on the surface of cells derived from oral SCC patient specimens, and together regulate adhesion and matrix remodelling [115].

### Angiogenesis

**Fig. 3:**
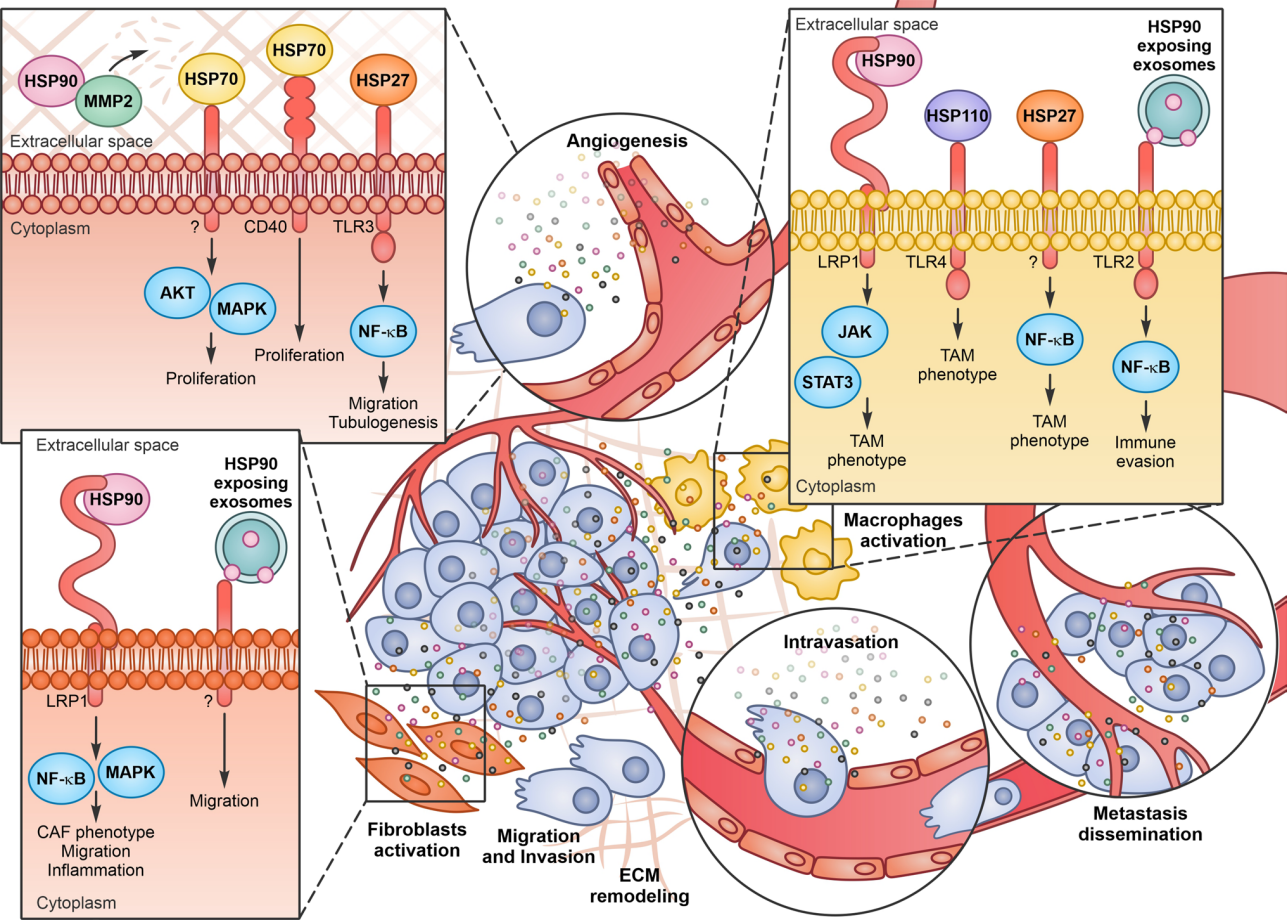
Extracellular Heat Shock Proteins (eHSPs) activity in the tumor microenvironment (TME). eHSPs can interact with different receptors on endothelial cells and induce angiogenesis, on fibroblasts and on macrophages and, activate them in cancer-associated fibroblasts (CAFs) and tumor-associated macrophages (TAMs) respectively. As a final result, eHSPs induce metastasis dissemination fuelling cancer progression

Angiogenesis, the process whereby new blood vessels develop from a pre-existing vascular network, is essential for normal organs, as well as for tumours, to establish a blood supply that satisfies their demand for oxygen and nutrients and accomplishes other metabolic functions. Hypoxia is a key driver of tumour angiogenesis and hypoxic cancer cells secrete vascular endothelial growth factor A (VEGFA), which initiates tumour angiogenesis binding to VEGF receptor 2 (VEGFR2) expressed on the endothelial cells of neighbouring blood vessels. In addition to VEGFA, others factors participate in this process including FGFα and β, platelet-derived growth factor (PDGF), TNFα, Angiopoietin1, MMPs, PA, TGFα and different interleukins as IL-1, IL-6 and IL-8. VEGFA, with the help of these pro-angiogenic molecules, induces the motility of endothelial cells and the remodelling of surrounding extracellular matrix leading to a tumour vascular network that is actively growing and infiltrative [116]. Extracellular chaperones, secreted not only by cancer cells but also by endothelial cells, may modulate angiogenesis (Fig. 3). Indeed, eHSP90α, once secreted in the medium, stabilizes MMP2 and favours the transmigration and tube formation of endothelial cells in vitro and in vivo. In a melanoma mouse model, blocking with neutralizing antibodies HSP90α, but not HSP90β, leads to a dose-dependent decrease in MMP2 activity, blood vessel density and tumour growth [117]. GRP78, the ER member of the HSP70 family, has been found secreted by colorectal and prostate carcinoma cells resistant to the antineoplastic agent bortezomib, a proteasome inhibitor with antiangiogenic activity. eGRP78 potently inhibits the pro-apoptotic activity of bortezomib on endothelial cells inducing the ERK/AKT pro-survival pathways and sustains angiogenesis [118]. eHSP27 was shown to promote angiogenesis through TLR3-dependent calcium entry and NF-κB activation in endothelial cells, which induce VEGF and IL-8 secretion. These factors produce autocrine and paracrine VEGFR2 activation, causing cell migration and tubulogenesis. In vivo experiments demonstrated that the depletion of HSP27 decreases vascularization and growth of breast and colon cancer cells in mouse and rat animal models and that the treatment with eHSP27 completely reverse this effect [119], highlighting a crucial role for eHSP27 in angiogenesis and cancer cell survival.

### Stromal cell activation

Fibroblasts, under normal condition, are devoted to tissue ECM maintenance. Once activated in the tumour microenvironment (mainly through TGFβ), fibroblasts change their structure and function, acquiring the phenotype of cancer-associated fibroblasts (CAFs). CAFs can produce cytokines and factors that stimulate tumour cells proliferation, migration and invasion and tumour immunosuppression, facilitating the invasive potential of cancer cells. Different reports suggest that eHSPs released by both cancer and stroma cells can modulate and activate fibroblasts, favouring cancer progression (Fig. 3). eHSP90α is observed to promote prostate fibroblast cell motility and to upregulate markers associated with a CAF-like phenotype, such as vimentin, αSMA, fibroblast activation factor (FAP) and tenascin C. eHSP90α, likely through LRP1 and the activation of NF-κB, induces fibroblasts to secrete inflammatory mediators as IL-6 and IL-8 in prostate cancer [120]. It has also been observed that the eHSP90α located on the external surface of breast cancer cell exosomes, induces fibroblast invasion that can be inhibited by treating cells with an eHSP90α blocking antibody [19].

Macrophages are important players in innate immunity and can polarize in M1 or M2 phenotypes depending on microenvironmental stimuli, such as cytokines, enzymes, and cell surface markers. M1 macrophages are involved in antitumor immunity and inflammatory responses characterized by the production of pro-inflammatory cytokines such as IL-6, IL-12, and TNFα. In contrast, the M2 phenotype is anti-inflammatory and pro-tumorigenic, and is characterized by the production of other types of cytokines such as IL-10 and TGFβ. Cancer cells recruit macrophages at the tumour site and activate them in tumour associated macrophages (TAM), that acquire a phenotype similar to M2 macrophages in the advanced stages of cancer progression. TAMs generate an immunosuppressive microenvironment and facilitate processes such as growth, angiogenesis and metastasis, producing cytokines, chemokines, and proteases. In pancreatic cancer cells, TAM express and secrete eHSP90α that binds to LRP1 and activates the Janus kinase (JAK) 2-STAT3 pathway, leading to cancer progression [121]. In lung cancer patients, a strong correlation was observed also between the serum concentration of eHSP70 and the percentage of M2 polarized macrophages [122]. HSP110, which is HSP70-related chaperone [3], activates stromal macrophages and is present in the serum of patients with colorectal cancer. Once secreted by colorectal cell lines, binding of HSP110 to TLR4 induces macrophages to polarize toward an M2 phenotype, while HSP110 immunodepletion, reverts this effect [123]. eHSP27, released by cancer cells in the TME, induces monocytes to express different cytokines, as IL-10, IL-6, and proangiogenic factors, as prostaglandin E2, VEGFA, IL-8, IL-1β, and TNFα. eHSP27 also promotes the expression and release of monocyte chemotactic protein-1 (MCP-1), a potent chemotactic signal for monocytes. eHSP27 may also mediate the differentiation of circulating monocytes into TAM-like macrophages with immunosuppressive and proangiogenic phenotypes [124]. eHSP90α exposed on the surface of tumour released autophagosomes can induce TLR2–MyD88–NF-κB signalling cascade and stimulate CD4^+^ T cells to produce IL-6 that functions in an autocrine manner to promote the production of IL-10 and IL-21, which create a favourable environment to facilitate tumour growth and metastasis in a melanoma mouse model [125] (Fig. 3).

Myeloid-derived suppressor cells (MDSCs) are an immature myeloid cell population that expands in subjects affected by cancer and inhibits T cell-mediated anti-tumor immunity. HSP72 exposed on tumor-derived exosomes binds to TLR2 on MDSCs and triggers STAT3 signalling, promoting IL6 expression and immunosuppressive activity [55]. Of note, a peptide aptamer able to interact with HSP72 [126] inhibits MDSC activation and tumor growth and robustly potentiates chemotherapy efficacy in cancer mouse models [57].

Even if it is not part of the topic covered by the review, it is worth mentioning that HSPs exposed on tumor-derived exosomes may also induce anti-tumor immunity, for instance by recruiting and activating natural killer cells [14, 17].

## Conclusions and future directions

Intracellular chaperones are essential in eliciting the initiation, the development, and the recurrence of cancer, making their overexpression and cell addiction critical requisites in cancer evolution [127]. A number of experimental data point to an equally important role of extracellular HSPs in promoting and sustaining different hallmarks of cancer. eHSPs are actively secreted by cancer cells and by other cell populations in the tumour microenvironment in response to stressful conditions. Once released, eHSPs interact both with extracellular clients and with membrane receptors and tune the behaviour of cancer cells, endothelial cells, fibroblasts and macrophages, orchestrating a complex interaction network, ultimately fuelling cancer growth, migration, invasion, angiogenesis and immune escape.

The targeting of HSPs may represent an attractive strategy in cancer treatment. Unfortunately, HSPs inhibition may seriously impacts on normal cell homeostasis, causing important side effects. Indeed, despite the big effort in testing HSPs inhibitors in cancer pre-clinical models, none of them have yet been approved by the FDA for the treatment of cancer patients. This is due to the severe toxicities and, except for HSP90 inhibitors, for the lack of convincing anticancer activity because of compensatory changes in other HSPs [27]. Instead, the possibility to block extracellular chaperones may represent an attractive alternative. Treatments with eHSP cell‐impermeable chemical inhibitors (like geldanamicyn beads, the geldanamicyn derivative DMAG-N-oxide, the ganetespib derivative STA-12–7191) [66–68, 128] or with monoclonal antibodies blocking eHSPs [44, 48–50, 67, 111], were able to impair cancer cell invasion and metastasis dissemination in tumour pre-clinical models, without any effects of systemic toxicity.

Moreover, even if the eHSP pro-tumorigenic role has been widely demonstrated, some caution is needed; eHSPs have been first studied for their ability to facilitate antigen presentation, stimulating anti-cancer immunity and inducing tumour regression [41, 129, 130]. The ideal situation would be to uncouple pro- and anti-tumorigenic effects of extracellular chaperones and selectively inhibit their pathological activity. The growing interest in the field and the increasing amount of data regarding extracellular chaperone function and behaviour is depicting a complex scenario in which specific extracellular co-chaperones may drive eHSP functions, allowing to hypothesize that a selective inhibition is feasible.
